# Effects of glucose spraying and lactic acid bacteria inoculation applied to high-moisture alfalfa during pre- and post-harvest periods on silage fermentation and feed quality

**DOI:** 10.7717/peerj.20276

**Published:** 2025-12-18

**Authors:** Fatma Akbay

**Affiliations:** Department of Field Crops, Faculty of Agriculture, Malatya Turgut Ozal University, Malatya, Turkey

**Keywords:** Silage, Lactic acid bacteria inoculation, Alfalfa, Lucerna, Glucose doses, Low DM, Silage quality, NDF and ADF, Fermentation, Corelation

## Abstract

**Background:**

The management of alfalfa silage in livestock production systems is crucial for achieving high-quality silage and optimal livestock production. Alfalfa (*Medicago sativa* L.) is known to be difficult to ensile due to its high buffering capacity (BC), low water-soluble carbohydrate (WSC) content, and low dry matter (DM) content. Therefore, using biological or chemical additives may be beneficial in improving the silage quality of alfalfa, particularly when it is harvested at relatively low DM content and high protein levels. Lactic acid bacteria (LAB) and glucose are commonly used as silage additives to enhance the ensiling process and improve fermentation quality.

**Methods:**

This study aimed to estimate the effects of pre-harvest and post-harvest application of lactic acid bacteria inoculant (control, *L. plantarum,* and * L. citerum)* and glucose doses (0%, 3% and 6%) treatments on the chemical composition and fermentation profile of silage feed of alfalfa grown under field conditions in 2023. After 60 days of ensiling, silages were analyzed for fermentation and quality characteristics.

**Results:**

Significant two-way and three-way interactions were observed among harvest, inoculant, and glucose dose for several parameters, including pH (T0, T60), dry matter ratio (DMT0, T60) content, and lactic acid bacteria (LAB T0) counts and acid detergent lignin (ADL) content. These interactions indicate that the effect of treatment varied depending on their combinations. The lowest initial pH(T0) was recorded in the pre-harvest combination *L. plantarum* with 0% glucose (*p* < 0.001). The silage pH(T60) value the lowest (4.91) was observed in 6% glucose combined with *L. plantarum*, while the highest pH was found in the control group with %0 glucose (*p* < 0.02). The highest initial DM(T0) content (25.78%) was achieved with the pre-harvest combination *L. citerum* + 3% glucose (*p* < 0.02). The highest silage DM (T60) content was noted with *L. citerum* (24.00%) and *L. plantarum* (23.20%) applied pre-harvest (*p* < 0.001). ADL content was recorded at its lowest value in the pre-harvest *L. citerum* with 3% glucose added (*p* < 0.03). The timing of harvest also had a significant effect on the quality parameters of silage (*p* < 0.001). Yeasts (T60) count and ADF content was lower in the pre-harvest treatments and crude protein content was higher in the pre-harvest (*p* < 0.01). Increasing glucose dose from 0% to 6% increased DM content, crude protein content and LAB count (*p* < 0.001).

**Conclusion:**

The best results for low pH and improved fermentation stability were achieved with 6% glucose and *L. plantarum* applied 24 h before harvest, which is recommended for practical use under field conditions. To suppress the yeast population, 3% and 6% glucose contents of *L. citerum* pre-harvest can be used alternatively.

## Introduction

Alfalfa (*Medicago sativa* L.), also known as “lucerne”, is an important legume forage crop which is widely used in livestock feed, especially as hay or green forage and, in some cases, as silage. Silage making from alfalfa requires careful management and know-how to prevent spoilage of feed while maintaining its nutritional values. However, difficulties encountered in this process can affect both production efficiency and the quality of the final product. Alfalfa is difficult to ensile due to its high buffering capacity (BC), low water soluble carbohydrate (WSC) and low dry matter (DM) content. It is usually wilted for hours or for days on the field after mowed, which causes additional costs and delays for regrowth of alfalfa crops. Therefore, the usage of biological or chemical additives may be beneficial (Zielińska, Fabiszewska & Stefańska, 2015) for achieving a satisfactory silage quality.

Generally, adding high WSC containing preparations to the silage or increasing the DM content may restrict butyric acid (BA) fermentation, which is one of the undesirable volatile acid components in silage, while lactic acid (LA) fermentation is increased. In other words, water-soluble carbohydrates such as glucose, fructose, sucrose and fructans are quantitatively the most important substrates for fermentation in cool-season crops (Ohyama, Masaki & Hoshino, 1973; McDonald, Edwards & Greenhalgh, 1991). Glucose can initially compensate for the loss of WSC caused by undesirable microorganisms (yeast, mould and aerobic bacteria), increase LA production and be effective in maintaining a sufficient amount of WSC in the medium for LAB growth. Zhang, Kawamoto & Cai (2010) reported that pH, acetic acid, butyric acid, and propionic acid decreased and lactic acid production increased with increasing glucose doses added to silages made from *Hordeum vulgare* L. and *Panicum maximum* Jacq. Similarly, Li et al. (2014) reported that the addition of glucose, molasses, sucrose and cellulose was effective in improving the fermentation quality of wheat silage. In many of these studies, sugar addition was mixed directly with the forage to be ensiled. Almost all studies on this subject were carried out under laboratory conditions, whereas “practical, effective, and economic” applications should be investigated under field conditions as well as laboratory conditions. Instead of inoculating the LAB and application of high doses of sugar just before silage making, it may be very useful to make them at least 24 h before ensiling and directly on the plant in the field and using very low sugar doses. In other words, it would be more rational to focus on the applicability of sugar or LAB applications under field conditions and to search for ways to facilitate the direct ensilability of alfalfa.

It is hypothesized that pre-harvest and post-harvest applications of different glucose doses combined with lactic acid bacteria inoculants would significantly enhance the fermentation quality and nutritional value of low DM alfalfa silage.

The objective of this study is to determine the most effective combination of glucose dose and LAB inoculant applied either pre-harvest or post-harvest in improving fermentation characteristics, microbial stability, and feed quality of alfalfa silage.

## Materials & Methods

Alfalfa obtained from the farmers field as second cut was inoculated with LAB in the pre-harvesting (24 before hours harvest) and post-harvesting (immediately after harvest) periods. The LAB strains used as inoculant and their strain numbers as follows; *Leuconostoc citerum* (*L. citerum*; L-70-6-1), *L. plantarum. Leuconostoc citerum and Lactobacillus plantarum* isolated from Turkiye grassland flora under a project supported by the Turkiye Scientific and Technical Research Organization (TUBITAK) were used as microbial inoculants. *Leuconostoc citerum and Lactobacillus plantarum were* regenerated in MRS (De Man, Rogosa ve Sharpe) broth in 400 ml bottles by incubation at 37 °C for 48 h. Cell densities were determined by cultivation on MRS agar medium.

### Silage preparation

For the pre-harvest applications, alfalfa at the 10% flowering period (DM about %20) was theoretically inoculated with a density of 10^8^ cfu/g fresh weight with two bacterial inoculants and three glucose doses (%0, 3 and 6) were applied prior to 24 h from cutting on the field. For pre-harvest, field inoculations were conducted in three replications, with a 3-meter interval maintained between each application to minimize the contamination risk. Fresh forage yield was assessed by cutting 2 m^2^ of alfalfa, which was weighed immediately prior to inoculation. Application dosages were subsequently calculated, and inoculation was performed using an atomizer for spraying.

For post-harvest, plant material was chopped into 2–4 cm fragments and separated into groups. Each strain and glucose was added to 4,000 g of fresh alfalfa plant material at a theoretical concentration of 10^8^ cfu/g ensuring thorough mixing by hand in sterile gloves.

Alfalfa materials were ensiled into plastic vacuum packages weighing 500 ± 40 g, with three replications conducted for all sampling times. A vacuum sealer was employed to remove 99.9% of O_2_ from the silage bags.

### Microbiological and chemical analysis

To monitor the microbial growth and pH changes for the first T_0_: before ensiling, and final silage T_60_: 60 days after ensilage. For T_60_ openings, two harvest times (pre-pro harvest), three inoculants (control, *L. citerum, L. plantarum),* three glucose doses (%0, 3 and 6) and three vacuum silages for each application (2 × 3 × 3 × 3 = 54 silage packets) were made separately. After the ensiling period, the silage was opened, and extracts were obtained through filtration using Whatman 54 filter paper (Whatman, Florham, NJ, USA).

The pH of the silage extracts was measured immediately following filtration to assess the acidity resulting from the fermentation process. Microbial counts were conducted using ten-fold serial dilutions. The enumeration of lactic acid bacteria was performed by pour plating on MRS agar with a double overlay for anaerobic conditions, followed by incubation at 36 °C for 48 to 72 h. Enterobacteria were quantified using pour plating on violet red bile glucose agar (VRBD) with a single overlay, and the plates were incubated at 36 °C for 18 h. Yeast and mold counts were assessed by pour plating on malt extract agar (MEA) acidified to pH 4 with lactic acid, using a single overlay and incubating at 32 °C for 48 h. The dry matter (DM) content of both fresh forage (designated as T0) and the resulting silage (designated as T60) was determined by drying samples at 70 °C in a forced-air oven for 48 h. After 60 days of ensiling, the silages were analyzed for several parameters, including pH, neutral detergent fiber (NDF), acid detergent fiber (ADF) ash crude protein (CP). Nitrogen (N) content was measured using the Kjeldahl method, with crude protein content calculated as N × 6.25 (AOAC, 1990) method. The cell wall fiber components, including NDF, ADF and ADL, were analyzed according to the methodology described by Van Soest, Robertson & Lewis (1991).

Dry matter digestibility (DMD), dry matter intake (DMI), and relative feed value (RFV) of silages were calculated by the following formulas developed by Van Dyke & Anderson (2000). DMD values were used to calculate the RFV.

DMD (%) = 88.9−(0.779 × ADF %)

DMI (%) = 120/NDF %

RFV = DMD %× DMI %× 0.775

### Statistical analysis

A factorial ANOVA model was used to analyse the effects of harvest time, glucose dose and inoculation type as fixed effects and their interactions. Repeats were considered as random effects. JMP statistical software was used for all analyses and mean comparisons were performed using the LSD test at a significance level of *p* < 0.05.

## Results

### Effects of harvest, inoculant, and glucose dose on ph, dry matter content, and microbial population of fresh alfalfa material before ensiling (T0)

Table 1 shows the effect of glucose doses and inoculant treatments on microbial population, pH levels, and dry matter (DM) content of fresh material before and after harvest. pH (T_0_) values were statistically significantly affected by the two-way interactions of Harvest x Inoculant, Harvest x Glucose, Glucose x Inoculant, and the three-way interaction of Harvest x Glucose x Inoculant (*p* < 0.05). Harvest x Glucose x Inoculant interaction significantly affected pH (T_0_) (*p* < 0.001), and the highest pH(T_0_) value of 6.60 was determined at post-harvest *L. plantarum* and 6% glucose dose, while the lowest pH(T_0_) value of 6.30 was determined at pre-harvest *L. plantarum* and 0% glucose dose.

**Table 1:** Effects of harvest, inoculant, and glucose dose on pH, dry matter content, and microbial population of fresh alfalfa material before ensiling (T0).

**Harvest**	**Inoculant**	**Glucose** **Doses (%)**	**pH** **(T**_**0**_)	**DM** **(T**_**0**_)	**LAB** **(T**_**0**_)	**Enterobacteria (T**_**0**_)	**Yeast** **(T**_**0**_)
**Pre-Harvest**	**Control**	**0**	6.41dfe	22.22ghı	2.29 g	5.26	6.88cd
		**3**	6.35 h	22.28ghı	4.17f	5.56	7.84abc
		**6**	6.37fgh	23.64cdef	6.14b	4.75	8.72a
	***L. plantarum***	**0**	6.30ı	25.23ab	2.44 g	5.74	8.20ab
		**3**	6.39efg	25.50ab	5.82bc	5.60	7.46bcd
		**6**	6.38fgh	24.79abc	4.80def	5.93	6.81cd
	***L. citerum***	**0**	6.43cd	24.44bcd	4.31f	5.71	8.79a
		**3**	6.39efg	25.57ab	6.14b	5.55	7.15bcd
		**6**	6.31ı	25.78a	6.04bc	5.73	7.06bcd
**Post-Harvest**	**Control**	**0**	6.36gh	22.13hı	4.34f	6.13	7.35bcd
		**3**	6.36gh	22.09ıj	4.59ef	6.37	6.45d
		**6**	6.39efg	22.94efghı	6.07b	6.17	7.03cd
	***L. plantarum***	**0**	6.46c	20.86j	5.27cde	5.80	7.24bcd
		**3**	6.52b	23.95cde	5.59bc	6.23	6.91cd
		**6**	6.60a	23.40defg	5.44bcd	5.45	7.34bcd
	***L. citerum***	**0**	6.44cd	22.58fghı	5.70bc	5.45	6.84cd
		**3**	6.42de	23.09efghı	6.97a	5.34	6.89cd
		**6**	6.42de	23.36defgh	6.06b	5.33	6.85cd
**Harvest**							
**Pre-Harvest**			6.40 b	24.38a	4.68b	5.54	7.66a
**Post-Harvest**			6.44 a	22.71b	5.56a	5.81	7.00b
**Glucose Doses (%)**							
**0**			6.39 b	22.91b	4.06c	5.68	7.55
**3**			6.40 ab	23.75a	5.54b	5.78	7.12
**6**			6.41 a	23.98a	5.76a	5.56	7.30
**Inoculant**							
**Control**			6.37c	22.55b	4.60b	5.71	7.38
***L. plantarum***			6.44c	23.95a	4.89b	5.79	7.33
***L. citerum***			6.40b	24.13a	5.87a	5.52	7.26
***F* value**							
Harvest			0.001	0.001	0.001	0.241	0.01
Glucose			0.020	0.001	0.001	0.742	0.196
Inoculant			0.001	0.001	0.001	0.607	0.885
Harvest*Glucose			0.001	0.337	0.001	0.908	0.734
Harvest*Inoculant			0.001	0.001	0.528	0.064	0.459
Glucose*Inoculant			0.001	0.059	0.001	0.936	0.070
Harvest*Glucose*Inoculant			0.001	0.019	0.033	0.799	0.01

**Notes.**

Means within the same column with different letters are significantly different (*P* < 0.05).

T0, Before Ensiling; DM, Dry matter ratio (%); LAB, lactic acid bacteria count.

Pre-harvest: treatments were applied 24 h before harvest.

Post-harvest: treatments were applied after harvesting.

Lactic acid bacteria count, enterobacteria count and yeast count (log_10_ cfu/g fresh material).

According to the research findings, both Harvest × Inoculant (*p* < 0.001) and Harvest x Glucose x Inoculant interactions were found to be statistically significant (*p* < 0.05) on dry matter (DMT_0_) content. When considering the interaction between harvest and inoculant, the highest dry matter (%DM) contents were determined in *L. plantarum* and *L. citerum* inoculants applied pre-harvest (25.17% and 25.26%, respectively). All other applications were statistically similar and did not show a significant difference when compared to these two applications (*p* < 0.001) (Table 2). Harvest x Glucose x Inoculant interaction significantly affected DM(T_0_) (*p* < 0.001), and the highest DM(T_0_) content of 25.78% was determined at pre-harvest *L. citerum* and 3% glucose dose. The lowest DM (T_0_) contents were found to be 20.86% for post-harvest *L.plantarum* inoculant and 0% glucose dose. This indicates that both pre-harvest applications and the combined effects of the inoculant and glucose doses applied play a decisive role in the dry matter accumulation of silage.

**Table 2:** Effects of harvest x glucose doses, harvest x inoculant, glucose doses x inoculant interactions on some silage parameters.

		**pH** **(T**_**0**_)	**DM** **(T**_**0**_)	**LAB** **(T**_**0**_)	**pH** **(T**_**60**_)	**DM** **(T**_**60**_)	**ADL**
Harvest*Glucose							
**Harvest**	**Glucose Doses(%)**						
Pre-Harvest	0	6.38c	23.96	3.02c	5.21	21.75	6.06bc
	3	6.38c	24.44	5.37ab	5.14	22.81	5.76c
	6	6.35d	24.73	5.66a	4.97	23.52	5.73c
Post-Harvest	0	6.42b	21.86	5.10b	5.30	19.67	6.07bc
	3	6.43b	23.04	5.71a	5.15	21.58	6.94a
	6	6.47a	23.23	5.86a	5.03	21.66	6.54ab
Harvest*Inoculant							
**Harvest**	**Inoculant**						
Pre-Harvest	Control	6.38c	22.71b	4.20	5.25	20.88b	5.98
	*L. plantarum*	6.36d	25.17a	4.36	4.96	23.20a	5.74
	*L. citerum*	6.38c	25.26a	5.49	5.11	24.00a	5.83
Post-Harvest	Control	6.37cd	22.39b	5.00	5.35	20.59b	6.69
	*L. plantarum*	6.53a	22.74b	5.43	5.03	20.99b	6.21
	*L. citerum*	6.43b	23.01b	6.24	5.10	21.34b	6.65
Glucose*Inoculant							
**Glucose Doses (%)**	**Inoculant**						
0	Control	6.38e	22.17	3.32f	5.50a	19.42	6.09
	*L. plantarum*	6.38e	23.05	3.86ef	5.08cde	21.44	5.66
	*L. citerum*	6.44c	23.51	5.00d	5.19c	21.48	6.45
3	Control	6.36f	22.18	4.38e	5.34b	20.76	6.56
	*L. plantarum*	6.46b	24.72	5.70bc	4.99ef	22.82	6.15
	*L. citerum*	6.41d	24.33	6.55a	5.11cd	23.02	6.32
6	Control	6.38e	23.29	6.11ab	5.07de	22.02	6.35
	*L. plantarum*	6.49a	24.09	5.12cd	4.91f	22.23	6.10
	*L. citerum*	6.37ef	24.57	6.05ab	5.02def	23.52	5.96

**Notes.**

Means within the same column with different letters are significantly different (*P* < 0.05).

T0, Before Ensiling; T60, Maturing silage; DM, Dry matter ratio (%); LAB, lactic acid bacteria count; ADL, acid detergent lignin.

Pre-harvest: treatments were applied 24 h before harvest.

Post-harvest: treatments were applied after harvesting.

Lactic acid bacteria count (log_10_ cfu/g fresh material).

In the triple interaction, the highest LAB count was determined in the post-harvest *L. citerum* inoculant and 3% glucose dose treatment, while the lowest LAB count was determined in the pre-harvest control treatment. The count of enterobacteria was not statistically affected by harvest, glucose dose, and inoculant treatments. Glucose doses and inoculant applications did not statistically affect the yeast count of fresh material. It was determined that harvest and harvest x glucose x inoculant interaction statistically affected the count of yeasts. According to the harvest*inoculant*glucose dose application, the lowest yeast count was detected in the post-harvest control application and 3% glucose dose application (*p* < 0.01).

### Effects of harvest timing, inoculant type, and glucose dose on fermentation parameters and microbial population of alfalfa silage after 60 days (T60)

Microbial population count, dry matter content and pH value of ensiling silages with different treatments after 60 days are shown in Table 3. pH values ranged from 4.91 to 5.50, with Glucose x Inoculant interactions having a particularly significant effect on pH values (*p* < 0.02). The highest pH value was observed in the 0% glucose + control application, while the lowest pH value was detected in the 6% glucose *+ L. plantarum* combination (4.91), indicating that the interaction between the inoculant and glucose rapidly stabilised the fermentation process (Table 2).

**Table 3:** Effects of harvest timing, inoculant type, and glucose dose on fermentation parameters and microbial population of alfalfa silage after 60 days (T60).

**Harvest**	**Inoculant**	**Glucose doses (%)**	**pH** **(T**_**60**_)	**DM** **(T**_**60**_)	**LAB** **(T**_**60**_)	**Enterobacteria (T**_**60**_)	**Yeast** **(T**_**60**_)
**Pre-Harvest**	**Control**	**0**	5.37	19.77	1.65	4.12	4.36
		**3**	5.32	20.55	2.75	3.53	4.37
		**6**	5.06	22.32	4.26	3.44	4.27
	***L. plantarum***	**0**	5.08	23.16	1.58	4.30	4.40
		**3**	4.97	23.13	3.85	4.25	3.96
		**6**	4.83	23.31	4.79	3.39	3.90
	***L. citerum***	**0**	5.19	22.32	1.96	3.68	4.29
		**3**	5.13	24.76	3.67	3.11	3.82
		**6**	5.00	24.93	4.84	3.52	3.56
**Post-Harvest**	**Control**	**0**	5.62	19.07	2.37	3.46	5.80
		**3**	5.36	20.97	2.90	3.18	5.26
		**6**	5.07	21.72	4.96	3.28	5.09
	***L. plantarum***	**0**	5.08	19.33	2.23	3.03	4.82
		**3**	5.00	22.50	3.15	3.08	4.78
		**6**	4.99	21.14	4.45	2.97	4.29
	***L. citerum***	**0**	5.20	20.63	1.67	3.23	4.65
		**3**	5.08	21.28	3.49	3.01	4.28
		**6**	5.03	22.11	3.71	2.46	4.05
**Harvest**							
Pre-Harvest			5.10	22.70a	3.26	3.71a	4.10b
Post-Harvest			5.16	20.97b	3.22	3.08b	4.78a
**Glucose Doses (%)**							
**0**			5.26a	20.71b	1.91c	3.64	4.72
**3**			5.14b	22.20a	3.30b	3.36	4.41
**6**			5.01c	22.59a	4.50a	3.18	4.19
**Inoculant**							
**Control**			5.30a	20.73b	3.15	3.50	4.85a
***L. plantarum***			4.99c	22.10a	3.34	3.50	4.36ab
***L. citerum***			5.11b	22.67a	3.22	3.17	4.11b
**Significance (*p*-value)**							
Harvest			0.055	0.001	0.808	0.024	0.001
Glucose			0.001	0.001	0.001	0.375	0.117
Inoculant			0.001	0.001	0.708	0.504	0.015
Harvest*Glucose			0.479	0.448	0.329	0.908	0.931
Harvest*Inoculant			0.296	0.001	0.083	0.668	0.416
Glucose*Inoculant			0.02	0.233	0.339	0.968	0.993
Harvest*Glucose*Inoculant			0.223	0.085	0.559	0.834	0.933

**Notes.**

Means within the same column with different letters are significantly different (*P* < 0.05).

T60, Maturing silage; DM, Dry matter ratio (%); LAB, lactic acid bacteria count.

Pre-harvest: treatments were applied 24 h before harvest.

Post-harvest: treatments were applied after harvesting.

Lactic acid bacteria count, enterobacteria count and yeast count (log_10_ cfu/g silage).

DM (T_6__0_) contents ranged from 19.67% to 24.00%, and the interaction between harvest and inoculant had a significant effect on DM contents, as shown in Tables 3 and 4. According to Table 2, the highest DM content was obtained with the application of *L. citerum* (24.00%) and *L. plantarum* (23.20%) pre-harvest. Other treatments were statistically similar.

**Table 4:** Chemical composition and relative feed value of alfalfa silage as affected by harvest timing, inoculant type, and glucose dose.

**Harvest**	**Inoculant**	**Glucose doses (%)**	**CP**	**NDF**	**ADF**	**ADL**	**RFV**
**Pre-Harvest**	**Control**	**0**	20.79	34.44	24.50	5.98cde	188.74
		**3**	22.65	34.36	23.71	5.83cde	190.93
		**6**	22.56	35.23	23.01	6.12cde	187.55
	***L. plantarum***	**0**	22.54	34.68	22.98	5.55de	190.70
		**3**	21.34	33.74	24.78	6.17cde	193.61
		**6**	23.31	32.97	22.32	5.49de	204.77
	***L. citerum***	**0**	20.63	34.19	25.15	6.65abc	188.73
		**3**	21.00	34.11	21.05	5.27e	197.93
		**6**	23.09	32.25	21.71	5.57de	215.11
**Post-Harvest**	**Control**	**0**	18.71	34.62	23.53	6.20cde	189.87
		**3**	19.92	34.13	27.46	7.30ab	185.10
		**6**	20.39	35.08	24.61	6.57abc	187.10
	***L. plantarum***	**0**	20.44	34.33	23.77	5.77cde	192.60
		**3**	21.27	34.65	23.14	6.13cde	191.10
		**6**	20.13	33.41	25.48	6.71abc	193.25
	***L. citerum***	**0**	19.41	34.22	24.91	6.25cde	190.66
		**3**	20.21	34.76	25.92	7.37a	185.39
		**6**	21.06	35.50	25.63	6.34bcd	181.19
**Harvest**							
**Pre-Harvest**			21.99a	34.00	23.25b	5.84b	195.34
**Post-Harvest**			20.17b	34.52	24.24a	6.52a	188.47
**Glucose Doses (%)**							
**0**			20.42b	34.41	24.14	6.07	190.21
**3**			21.06ab	34.29	24.34	6.35	190.67
**6**			21.76a	34.07	23.79	6.13	194.83
**Inoculant**							
**Control**			20.83	34.64	24.47	6.33	188.22
***L. plantarum***			21.50	33.96	23.74	5.97	194.34
***L. citerum***			20.90	34.17	24.07	6.24	193.16
**Significance (*p*-value)**							
Harvest			0.001	0.558	0.009	0.001	0.250
Glucose			0.002	0.952	0.762	0.345	0.780
Inoculant			0.119	0.814	0.628	0.179	0.668
Harvest*Glucose			0.208	0.853	0.116	0.018	0.506
Harvest*Inoculant			0.381	0.810	0.380	0.657	0.626
Glucose*Inoculant			0.120	0.941	0.475	0.373	0.977
Harvest*Glucose*Inoculant			0.306	0.958	0.192	0.034	0.893

**Notes.**

Means within the same column with different letters are significantly different (*P* < 0.05).

CP Crude protein Ratio NDF neutral detergent fiber ADF acid detergent fiber ADL acid detergent lignin RFV relative feed value

Pre-harvest: treatments were applied 24 h before harvest.

Post-harvest: treatments were applied after harvesting.

Table 3 shows that glucose doses statistically affected the count of LAB, and as the glucose dose increased, the count of LAB increased significantly. Post-harvest applications were found to have a lower count of enterobacteria. But the count of yeasts was lower in the pre-harvest treatments. Glucose doses did not affect enterobacteria and yeast counts in a statistically significant way. *L. citerum* inoculant significantly reduced yeast counts when compared to control and *L. plantarum*. As shown in Table 3, *L. citerum* was the most effective inoculant. Mold could not be determined on the 60th day of silage.

### Chemical composition and relative feed value of alfalfa silage as effected by harvest timing, inoculant type, and glucose dose

Table 4 shows the chemical composition of alfalfa silage. Although the interaction effects in Table 4 were not statistically significant, noticeable mean differences among treatments were still observed. In the present study, NDF contents of harvest x inoculant x glucose doses interaction from 32.25 to 35.50% and no statistically significant difference occurred. ADF and ADL value was lower in pre-harvest treatments. A significant harvest x inoculant x glucose doses interaction was observed for ADL content (*p* < 0.03). The lowest ADL value was recorded in the *L. citerum* and 3% glucose treatment pre-harvest, while higher values were observed in the post-harvest *L. citerum* and 3% glucose treatment. Combining glucose supplementation and bacterial inoculant before harvest can effectively reduce the lignin content. The average RFV value was found to be 191.91 RFV value was not statistically affected by the treatments.

Post-harvest (20.17%) exhibited significantly lower CP content compared to pre-harvest (21.99%) of silage alfalfa (*p* < 0.001). Glucose doses (*p* < 0.0021) significantly affected CP content. The highest protein content was determined at 6% glucose dose with 21.76% and the lowest was determined in the control group. Inoculation treatment and Harvest*Glucose*Inoculant interaction did not affect protein content (Table 4).

### Correlogram of microbial and chemical traits in alfalfa silage

**Figure 1:**
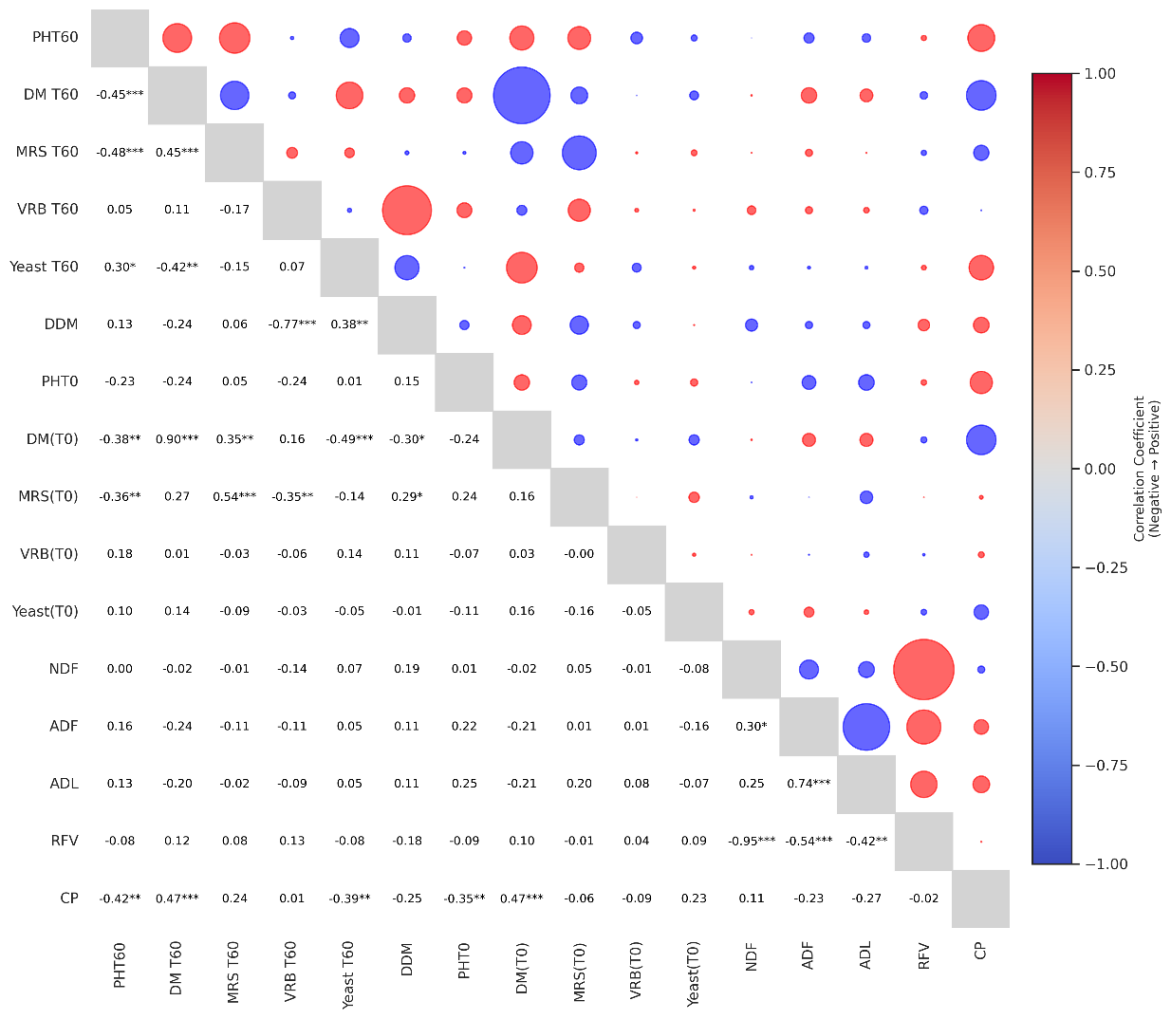
Correlogram of microbial and chemical traits in alfalfa silage. (T0), fresh material; (T60), mature silages; DM, Dry matter ratio; DDM, dry matter recovery; MRS, lactic acid bacteria count; VRB, enterobacteria count; CP, crude protein ratio; NDF, neutral detergent fiber; ADF, acid detergent fiber; ADL, acid detergent lignin; RFV, relative feed value. Red circles indicate positive correlations, while blue circles indicate negative correlations. The size of each circle represents the strength of the correlation. Asterisks indicate the level of statistical significance: ^∗^*p* < 0.05, ^∗∗^*p* < 0.01, ^∗∗∗^*p* < 0.001.

Figure 1 shows the correlation matrix between various chemical and microbial parameters affecting the quality of alfalfa silage after 60 days of fermentation. Positive correlations are indicated by red circles and negative correlations by blue circles, with the size of the circles proportional to the strength of the relationship. The lower triangle shows Pearson correlation coefficients with statistical significance levels (^∗^*p* <  0.05, ^∗∗^*p* < 0.01, ^∗∗∗^*p* < 0.001). In particular, a strong positive correlation was observed between dry matter (DM) and dry matter conservation (DDM), emphasizing the contribution of moisture control to dry matter conservation. In contrast, neutral detergent fiber (NDF) and acid detergent fiber (ADF) were negatively correlated with relative feed value (RFV), suggesting a trade-off between fiber content and digestibility. Similarly, a positive correlation was observed between crude protein (CP) and relative feed value (RFV). This suggests that feeds with higher protein content generally have higher feed quality. Negative correlations were also found between the enterobacteria parameter and pH, suggesting that especially enterobacteria species are suppressed in the acidic environment of fermentation. At day 60, a positive and statistically significant relationship (*r* = 0.30, *p* < 0.05) was determined between the yeast (T60) population and the final pH (T60) value of silage. This result indicates that yeast growth was higher in silage samples with higher pH levels. The fact that yeast species become more active in low acidic environments also supports this finding biologically. Insufficient acid formation during the fermentation process, pH not decreasing sufficiently and therefore yeast growth cannot be controlled may adversely affect the stability of silage. This finding shows that low pH is important not only for bacterial fermentation but also for the control of yeast populations. There was a moderate negative and statistically significant relationship (*r* = −0.39, *p* < 0.01) between crude protein content and yeast population at 60 days. This shows that yeast growth was suppressed in silage samples with high protein content. In other words, we can say that there was no dehydration in protein content in silages with low yeast growth. Probably, fermentation progressed more effectively in these samples and the acidic environment was better formed. No statistically significant relationship was observed between crude protein and enterobacteria population (T60) (*r* = 0.01, *p* < 0.93).

## Discussion

The presence of lactic acid bacteria (LAB) in the fresh material plays an important role in silage fermentation (Günaydın et al., 2023). Previous studies have shown that the minimum number of LAB in the plant should exceed 5.0 (log10 cfu TM) (Lin et al., 1992). The LAB count in fresh alfalfa material was 6.97 (log10 cfu/TM) in postharvest treatments and at 3% glucose dose and *L.citerum* inoculant, which met this requirement quite well. Soluble carbohydrates are important sources of energy for plant growth and metabolism (Wang et al., 2018). In both glucose dose treatments (3%, 6%), the number of LAB exceeded that required in fresh material. These results are consistent with Ohyama, Masaki & Hoshino (1973) and McDonald, Edwards & Greenhalgh (1991) who emphasized the importance of water soluble carbohydrates for LAB growth and fermentation efficiency.

The pH value is a key indicator to assess the fermentation quality of silage (Kung Jr et al., 2018) and a sufficient number of lactic acid bacteria is required to lower the pH value and obtain high quality silage (Ni et al., 2015) LAB inoculant is quite successful in pH reduction (Akbay, Günaydın & Kizilsimsek, 2025). However, it is seen that *L. plantarum* inoculant is more effective in pH reduction than *L. citerum*. Kızılşimşek et al. (2016) reported that the lactic acid production efficiency of *L. plantarum* inoculant was higher than *L. citerum*. The higher the lactic acid production efficiency, the faster the pH decreases. *L. plantarum* is homofermentative, while *L. citerum* is heterofermentative, which could be the reason for lower pH in *L. plantarum* since homofermentative produce more of lactic acid, and lactic acid is 10 to 12 times stronger than any of the other major acids to potentially reduce pH (Kung Jr et al., 2018). However, no organic acids were determined after the silage fermentation, which is a limitation of the present study. Similar to our study, many studies emphasized that *L. plantarum* inoculant was more effective in lowering the pH value (Liu et al., 2022; Wang et al., 2022). Moreover, glucose treatments significantly lowered the pH. This is consistent with many studies reporting that glucose addition increases lactic acid production and suppresses undesirable microbial activity (Shao et al., 2004; Zhang, Kawamoto & Cai, 2010; Li et al., 2014). However, the pH value was lower in the pre-harvest treatments in the starting material, indicating that the fermentation processes started earlier. On the other hand, lower pH was obtained in pre-harvest treatments as a result of fermentation. This is an indication of successful fermentation. The pH was 4.83 in pre-harvest silages treated with 6% glucose dose and *L. plantarum* inoculant. This value is quite good for alfalfa silage with low dry matter content.

Glucose application did not affect the number of enterobacteria and yeasts in fresh material and fermentation results. However, inoculant application, especially *L. citerum* inoculant, decreased the number of yeasts in silages, which is in line with many studies showing that inoculants improve aerobic stability by increasing lactic acid production and suppressing the growth of undesirable microorganisms (Keleş & Yazgan, 2005; Ogunade et al., 2018; Ertekin, 2023). Harvest treatments can be said to be partially effective on the number of yeast and enterobacteria at the end of fermentation. It can be said that the number of yeast and enterobacteria is less in pre-harvest applications, this situation indicates that the fermentation process starts earlier in pre-harvest applications, and pre-harvest applications are more effective in reducing the number of yeast in the fermentation result. Kara, Polat & Koç (2016) reported that especially the use of pre-harvest inoculants had positive effects on the microbial composition of silages, LAB counts increased in silages with inoculants compared to the control group, and no mold was detected in silages. Pre-harvest and post-harvest treatments significantly affected DM content. Pre-harvest treatments resulted in higher DM content, which is a critical factor for silage preservation and animal feed efficiency. This may be attributed to the post-harvest LAB inoculation and water use in glucose treatments reducing dry matter content.

No significant difference was observed in NDF, ADF, ADL and RFV between inoculant treatments, indicating that the structural carbohydrate content of silage remained constant and was not affected by inoculants. Similarly, Kızılşimşek et al. (2016), Guo et al. (2018) reported that NDF and RFV values were not affected. Moreover, Ertekin (2023) reported that CP, ADF, NDF, and RFV values were not affected by inoculant applications. On the other hand, many researchers reported that NDF, ADF contents decreased with inoculant application (Liu et al., 2016; Huo et al., 2021). This difference between the studies is thought to be due to the difference in DM content and plant species. On the other hand, NDF, ADF and ADL contents were slightly lower in pre-harvest treatments. The RFV value is higher, indicating better digestibility and uptake potential. These results are consistent with previous studies reporting improved silage quality with pre-harvest treatments and glucose supplementation (Zielińska, Fabiszewska & Stefańska, 2015).

## Conclusions

This study found that the interaction between glucose application, inoculant type, and harvest timing played a significant role in determining the fermentation quality of alfalfa silage. This underscores the importance of optimizing these factors together, rather than in isolation, when seeking to enhance silage quality in field conditions. As a result of the study, it was determined that application of 6% glucose dose and *L. plantarum* inoculant 24 h before harvest under field conditions improved silage fermentation and is therefore recommended.

##  Supplemental Information

10.7717/peerj.20276/supp-1 Supplemental Information 1 Effects of harvest, inoculant, and glucose dose on pH, dry matter content, and microbial population of fresh alfalfa material before ensiling (T0)

10.7717/peerj.20276/supp-2 Supplemental Information 2 Effects of harvest timing, inoculant type, and glucose dose on fermentation parameters and microbial population of alfalfa silage after 60 days (T60)

10.7717/peerj.20276/supp-3 Supplemental Information 3 Chemical composition and relative feed value of alfalfa silage as affected by harvest timing, inoculant type, and glucose dose

10.7717/peerj.20276/supp-4 Supplemental Information 4 Correlogram of microbial and chemical traits in alfalfa silage

10.7717/peerj.20276/supp-5 Supplemental Information 5 Core findings and methodology of the study, providing a concise visual overview of the experimental design and results

## Additional Information and Declarations

### Data Availability

The following information was supplied regarding data availability: The raw data is available in the Supplemental Files.
