# Supplementary figures and images for: Effects of glucose spraying and lactic acid bacteria inoculation applied to high-moisture alfalfa during pre- and post-harvest periods on silage fermentation and feed quality
Source: PeerJ. 2025 Dec 18;13:e20276. doi: 10.7717/peerj.20276 (PMC12718523; doi:10.7717/peerj.20276)

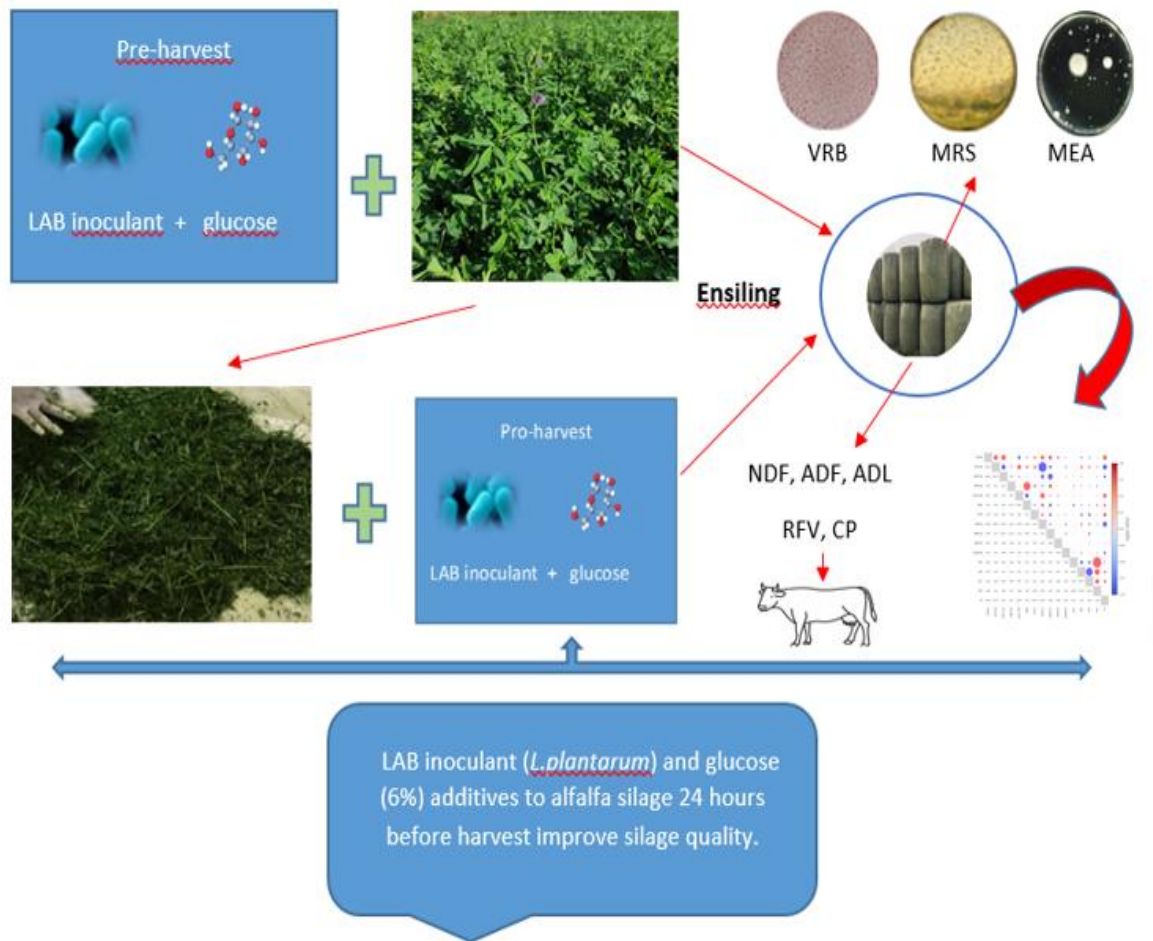

Supplement: Supplemental Information 5 [file peerj-13-20276-s005.pdf]
